# Synthesis and Chemical Characterisation of New Bis-Thieno [2,3-b]thiophene Derivatives

**DOI:** 10.3390/molecules15053329

**Published:** 2010-05-07

**Authors:** Yahia Nasser Mabkhoot

**Affiliations:** Department of Chemistry, Science of College, King Saud University, PO Box 2455, Riyadh-11451, Saudi Arabia; E-Mail: yahia@ksu.edu.sa

**Keywords:** thieno[2,3-b]thiophene, 2,5-dicarbohydrazide, 5-Amino-3-cyano(1,2,3)tria-zole-1-carbonyl

## Abstract

Using 3-methyl-4-phenylthieno[2,3-b]thiophene-2,5-dicarbohydrazide as synthon a series of new bis-heterocycles incorporating the thieno[2,3-b]thiophene nucleus was prepared and characterized.

## 1. Introduction

Thienothiophene derivatives represent important building blocks in organic and medicinal chemistry. They have been developed for different pharmaceutical purposes and have been tested as potential antitumor, antiviral, antibiotic, and antiglaucoma drugs, or as inhibitors of platelet aggregation [1,2,3,4,5,6]. On the other hand, hydrazone derivatives are reported to possess antimicrobial [7], antitubercular [8], anticonvulsant [9] and anti-inflammatory [10] activities.

The utility of hydrazides as key intermediates in the synthesis of several series of heterocyclic compounds and the broad spectrum of biological activities that have been reported for their cyclized products [11,12,13,14] has aroused interest in exploring the utility of hydrazides as versatile precursors for the synthesis of a variety of substituted heterocycles [15,16,17,18,19]. Several Schiff’s bases, hydrazones and hydrazides of isoniazid have shown good activity against tubercular, fungal and bacterial infections [20,21]. A number of hydrazide–hydrazone derivatives have been claimed to possess interesting antibacterial, antifungal, anticonvulsant, antiinflammatory, antimalarial and antituberculosis- activities [22]. Acid hydrazides can be considered as useful intermediates leading to the formation of several heterocycles such as pyrazole and triazoles. Pyrazole derivatives are a very interesting class of heterocyclic compounds that have remarkable pharmacological activities as antibacterial, antifungal, and hypoglycemic compounds, as tumor necrosis inhibitor, and in the treatment of thromboembolic disorders [23,24,25,26]. In continuation of these findings, we report herein the synthesis of some novel bis-heterocycles containing a thieno[2,3-b]thiophene moiety as a base unit which are of interest as potential biologically active compounds or pharmaceuticals. 

## 2. Results and Discussion

**Scheme 1:**
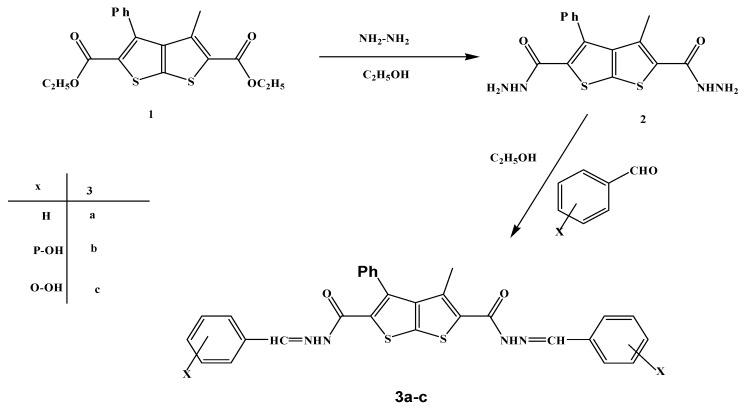
Reaction of 2,5-dicarbohydrazide **2** with aromatic aldehydes.

Diethyl 3-methyl-4-phenylthieno[2,3-*b*]thiophene-2,5-dicarboxylate (**1**) was prepared according to literature methods [27]. Next, the reaction of compound **1** with hydrazine hydrate in refluxing ethanol gave the bis-hydrazide **2** (Scheme 1). The IR spectrum of the latter revealed the appearance of three absorption bands at 3,304, 3,220, and 3,159 cm^–1^ due to NH_2_ and NH functions and its mass spectrum showed a peak corresponding to its molecular ion at m/z = 346 [M+].

Subsequent treatment of compound **2 **with appropriate aldehydes in refluxing ethanol yielded the corresponding hydrazones **3a-c **(Scheme 1). The structures of the latter products were established on the basis of the appearance of an NH absorption band in the 3,229–3,140 cm^-1^ region and a carbonyl function band in the 1665-1644 cm^-1^ region of their IR spectra, whereas their ^1^H-NMR spectra revealed the presence of a signal due to the -CH=N- proton in the 8.12–8.56 ppm region and a D_2_O exchangeable signal (NH) in the 9.98-10.75 ppm region.

**Scheme 2:**
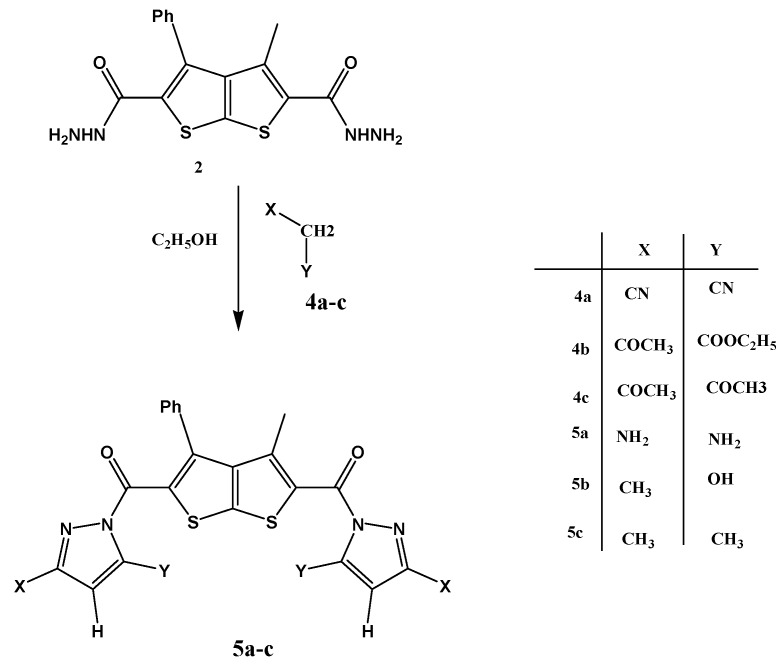
Reaction of 2,5-dicarbohydrazide **2** with active methelene derivatives **4a-c**.

The hydrazide derivative **2 **also reacted with active methylene derivatives**4a-c **to afford the corresponding pyrazolo derivatives **5a-c **(Scheme 2). The structures of compounds **5a-c **were in agreement with their spectral and analytical data. For example, the ^1^H-NMR spectrum of compound **5b** contained a new singlet at δ = 8.10 ppm, not present in the spectrum of the starting material, and attributed to the CH of the pyrazolo ring, and the mass spectra of **5a-c** contained molecular ion peaks at m/z = 478, 478, and 474, respectively, in agreement with their calculated masses.

**Scheme 3:**
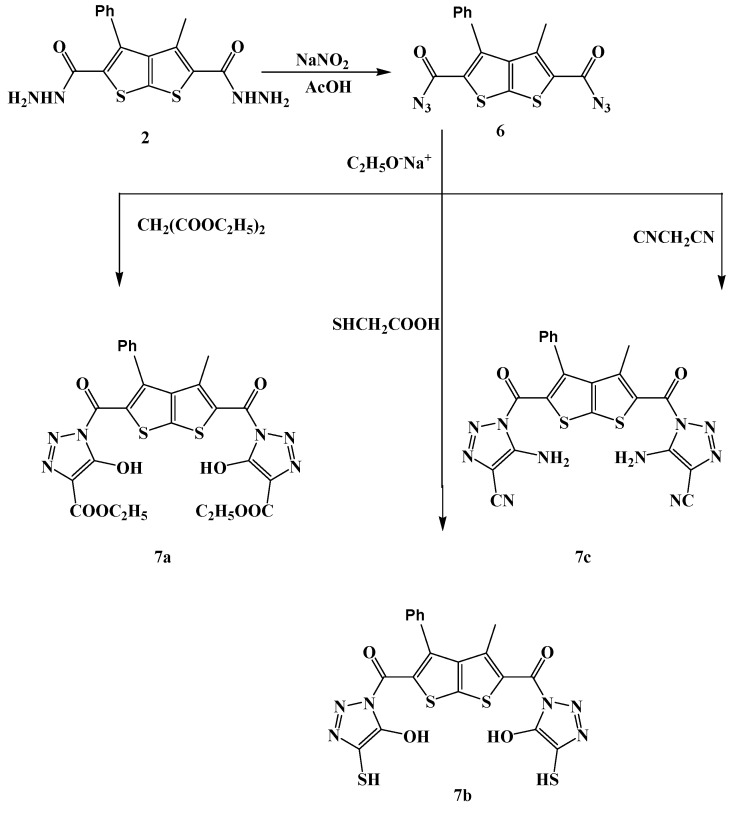
Reaction of 3-methyl-4-phenylthieno[2,3-b]thiophene-2,5-dicarbonyl diazide (**6**) with active methylene derivatives.

Treatment of compound **2 **with the sodium nitrite in acetic acid yielded the corresponding azide derivative **6 **(Scheme 3). Its IR spectrum and its ^1^H-NMR were free of NH and NH_2_ proton signals.

The reaction of compound **6** with active methylene derivatives in sodium ethoxide afforded the corresponding triazole derivatives **7a-c **(Scheme 3). The structures of the latter were deduced from their elemental analyses and spectral data. The ^13^C-NMR spectrum of **7c**, as an example, revealed fourteen carbon signals. Its ^1^H-NMR spectrum displayed singlets at δ 4.25 ppm attributable to the NH_2_ protons. Its IR spectrum revealed the appearance of an absorption band at 3,309 cm^–1^ due to the NH_2_ groups, in addition to the carbonyl absorption band at 1,685 cm^–1^. Its mass spectrum showed a peak corresponding to its molecular ion at m/z = 500 [M+].

## 3. Experimental

### 3.1. General

All melting points were measured on a Gallenkamp melting point apparatus. IR spectra were measured as KBr pellets on a Pye-Unicam SP 3-300 spectrophotometer. The NMR spectra were recorded on a Varian Mercury VX-300 NMR spectrometer. ^1^H-NMR (300 MHz) and ^13^C-NMR (75.46 MHz) were run in dimethylsulphoxide (DMSO-d_6_). Mass spectra were recorded on a Shimadzu GCMS-QP 1000 EX mass spectrometer at 70 eV. Elemental analysis was carried out on an Elementar Vario EL analyzer. Thieno[2,3-*b*]thiophene derivative **1** was prepared following a literature procedure [27]. 

*3-Methyl-4-phenylthieno[2,3-b]thiophene-2,5-dicarbohydrazide* (**2**). A mixture of compound **1** (3.74 g, 10 mmol) and hydrazine hydrate (1.0g, 20 mmol) in absolute ethanol (100 mL) was refluxed for 2 h. The separated white solid was filtered off and recrystallized from EtOH / DMF to give the title compound **2**. Yield: 87%; m.p. 204-206°C; IR (ν_max_): 3,304, 3,220, 3,159 (NH, NH_2_), 1639 (C=O) cm^‑1^; ^1^H-NMR: δ 1.85 (s, 3H, CH_3_), 4.39–4.50 (br. s, 4H, NH_2_, D_2_O exchangeable), 7.39–7.49 (m, 5H, ArH), 8.26 (s, 1H, NH), 9.41(s, 1H, NH); ^13^C-NMR: δ 14.4, 128.8, 130.2, 132.9, 134.5, 136.3, 138.8, 146.0, 162.1, 162.8, 171.3; MS m/z (%): 347 (M^+^ + 1, 94), 348 (M^+^ + 2, 66.7), 346 (M+, 100), 206.9 (25.3); Anal. calcd. for C_15_H_14_N_4_O_2_S_2_ (346.43): C, 52.01; H, 4.07; N, 16.17; S, 18.51. Found: C, 51.97; H, 4.11; N, 16.18; S, 18.47.

### 3.2. Reaction of 3-methyl-4-phenylthieno[2,3-b]thiophene-2,5-dicarbohydrazide (**2**) with aldehydes

A mixture of the hydrazide **2** (3.46 g, 10 mmol) and the appropriate aldehyde (20 mmol) in ethanol (50 mL) was refluxed for 4 h. The formed solid product was collected by filtration, washed with ethanol and dried. Recrystallization from the appropriate solvent afforded the corresponding hydrazone derivatives **3a-c**.

*Dibenzylidene-3-methyl-4-phenylthieno[2,3-b]thiophene-2,5-dicarbohydrazide *(**3a**). Yellowish solid; 77%; m.p. 295 °C (EtOH/DMF); IR ν_max_: 3,140 (NH), 1,657 (C=O) cm^-1^; ^1^H-NMR: δ 2.13 (s, 3H, CH_3_), 7.43-7.68 (m, 15H, ArH), 6.77(s, 2H, -CH=N-), 10.75 (br. s, 2H, NH, D_2_O-exchangable); ^13^C-NMR: δ 12.3, 123.7, 127.5, 128.7, 129.5, 131.6, 135.4, 136.7, 150.8, 159.3, 173.2; MS *m/z *(%) 523 (M^+^+1, 88.6%), 522 (M+, 100%), 207.9 (8.1%), 116 (8.4%), 62.9 (71.4.5%); Anal. calcd. for C_29_H_22_N_4_O_2_S_2_ (522.6): C, 66.64; H,4.24; N, 10.52; S, 12.27. Found: C, 66.70; H, 4.14; N, 10.70; S, 12.24.

*Bis(2-hydroxybenzylidene)-3-methyl-4-phenylthieno[2,3-b]thiophene-2,5-dicarbohydrazide* (**3b**). Yellowish solid; yield 80%; m.p. >300 °C (EtOH/ DMF); IR ν_max_: 3,304 (OH), 3,229 (NH), 1,644 (C=O) cm^-1^; ^1^H-NMR: δ 2.00 (s, 3H, CH_3_), 7.36-7.57 (m, 13H, ArH), 6. 85 (s, 2H, -CH=N ), 9.57 (br. s, 2H, NH,), 9.98(s, 1H, OH,), 11.7 (s, 1H, OH,); ^13^C-NMR: δ 12.3, 123.6, 127.3, 128.8, 129.5, 131.6, 135.4, 136.7, 150.8, 159.3, 172.8; MS *m/z *(%) 555 (M^+^+1, 88.6%), 554 (M+, 100%), 222 (9.4%), 161 (7.6%), 46.9 (44.7.5%); Anal. calcd. for C_29_H_22_N_4_O_4_S_2_ (554.6): C, 62.80; H,4.00; N, 10.10; S, 11.56. Found: C, 62.70; H, 4.14; N, 10.11; S, 12.44.

*Bis(4-hydroxybenzylidene)-3-methyl-4-phenylthieno[2,3-b]thiophene-2,5-dicarbohydrazide *(**3c**). Yellowish solid; yield 85%; m.p. >300 °C (EtOH/ DMF); IR ν_max_: 3,306 (OH), 3,229 (NH), 1,665 (C=O) cm^-1^; ^1^H-NMR: δ 2.00 (s, 3H, CH_3_), 7.33-7.67 (m, 13H, ArH), 6.86 (s, 2H, -CH=N-), 8.57 (br. s, 2H, NH), 9.99 (s, 1H, OH), 11.66 (s, 1H, OH); ^13^C-NMR: δ 12.3, 123.2, 126.3, 128.9, 131.5, 135.9, 136.7, 150.8, 159.3, 176.6; MS *m/z *(%) 555 (M^+^+1, 88.6%), 554 (M+, 100%), 222 (9.4%), 161 (7.6%), 46.9 (44.7.5%); Anal. calcd. for C_29_H_22_N_4_O_4_S_2_ (554.6): C, 62.80; H,4.00; N, 10.10; S, 11.56. Found: C, 62.70; H, 4.14; N, 10.11; S, 12.44.

### 3.3. Reaction of 3-methyl-4-phenylthieno[2,3-b]thiophene-2,5-dicarbohydrazide (**2**) with active methylene derivatives

A mixture of the hydrazide **2** (3.46 g, 10 mmol) and the appropriate malononitrile, ethyl acetoacetate or acetyl acetone **4a-c** (20 mmol) in ethanol (20 mL) was refluxed for 5 h. After cooling the obtained solid was collected by filtration, dried and crystallized from EtOH/DMF. 

*[5-(3,5-Diamino-pyrazole-1-carbonyl)-3-methyl-4-phenylthieno[2,3-b]thiophene-2-yl]-(3,5-diamino-pyrazol-1-yl)-methanone* (**5a**). Yellowish solid; yield 60%; m.p. >300 °C; IR ν_max_: 3,310–2,840 (2NH_2_), 1,670 (C=O) cm^-1^; ^1^H-NMR: δ 2.00 (s, 3H, CH_3_), 4.33 and 4.50 (br. s, 4H, 4NH_2_, D_2_O exchangeable), 7.40–7.43 (m, 5H, ArH), 6.67 (s, 2H, -2CH=C); ^13^C-NMR: δ 12.7, 89.7, 127.7, 128.3, 129.8, 135.1, 139.7, 142.2, 140.3, 144.1, 152.8, 174.0; MS *m/z *(%) 479 (M^+^+1, 88.6%), 478 (M+, 100%), 207 (30.7%), 76 (15.8%); Anal. calcd. for C_21_H_18_N_8_O_2_S_2_ (478.5): C, 52.71; H, 3.79; N, 23.42; S, 13.40. Found: C, 52.56; H, 3.65; N, 23.52; S, 13.28.

*[5-(5-Hydroxy-3-methyl-pyrazole-1-carbonyl)-3-methyl-4-phenylthieno[2,3-b]thiophene-2-yl]-(5-hydroxy-3-methyl-pyrazol-1-yl)-methanone* (**5b**). Yellowish solid; yield 60%; m.p. 296 °C; IR ν_max_: 3,310 (OH), 1,669 (C=O) cm^-1^; ^1^H-NMR: δ 2.00 (s, 3H, CH_3_), 2.43 (s, 6H, 2CH_3_), 7.42–7.43 (m, 5H, ArH), 6.10 (s, 2H, -CH=C), 13.1 (s, 2H, OH); ^13^C-NMR: δ 12.7, 13.9, 91.7, 127.7, 128.3, 129.8, 135.1, 139.7, 142.2, 143.2, 144.1, 152.8, 174.0; MS *m/z * (%) 479 (M^+^+1, 88.6%), 478 (M+, 100%), 207 (32.1%), 76 (11.8%); Anal. calcd. for C_23_H_18_N_4_O_4_S_2_ (478.5): C, 57.73; H, 3.79; N, 11.71; S, 13.40. Found: C, 58.00; H, 3.64; N, 11.70; S, 13.49.

*[5-(3,5-Dimethyl-pyrazole-1-carbonyl)-3-methyl-4-phenylthieno[2,3-b]thiophene-2-yl]-(3,5-dimethyl- yrazol-1-yl)-methanone* (**5c**). Colorless solid; yield 65%; m.p. >300 °C; IR ν_max_: 1,670 (C=O) cm^-1^; ^1^H-NMR: δ 1.91 (s, 3H, CH_3_), 1.96 (s, 6H, CH_3_), 2.00 (s, 6H, 2CH_3_) 7.41–7.42 (m, 5H, ArH), 6.10 (s, 2H, -CH=C); ^13^C-NMR: δ 12.7, 15.6, 92.0, 128.3, 129.8, 135.1, 139.7, 142.2, 140.2, 144.1, 146.8, 164,1 174.0; MS *m/z *(%) 475.6 (M^+^+1, 88.6%), 474.6 (M+, 100%), 207 (14.1%), 76 (11.8%), 46.9 (9.9%); Anal. calcd. for C_25_H_22_N_4_O_2_S_2_ (474.6): C, 63.27; H, 4.67; N, 11.81; S, 13.51. Found: C, 63.10; H, 4.68; N, 11.67; S, 13.49.

*3-Methyl-4-phenylthieno[2,3-b]thiophene-2,5-dicarbonyl diazide* (**6**). A mixture of compound **2** (3.74 g, 10 mmol) in acetic acid (30 mL) was treated with 10% sodium nitrite (2.76 g, 40 mmol) which was added dropwise at -5 °C with stirring for 1 h. The solid product was filtered off and recrystallized from ethanol. Colorless solid; yield 87%; m.p. 122°C; IR ν_max_: 1,678 (C=O) cm^-1^; ^1^H-NMR: δ 2.01 (s, 3H, CH_3_), 7.34–7.47 (m, 5H, ArH); ^13^C-NMR: δ 14.6, 128.4, 128.7, 129.0, 129.5, 129.7, 131.0, 131.3, 147.0, 164.1; MS *m/z *(%) 369 (M^+^+1, 88.6%), 368 (M+, 100%); Anal. calcd. for C_15_H_8_N_6_O_2_S_2_ (369.39): C, 48.90; H, 2.19; N, 22.81; S, 17.41. Found: C, 49.03; H, 2.30; N, 22.86; S, 17.42.

### 3.4. General procedure for the synthesis of compounds **7a-c**

A solution of Na (0.56 g, 20 mmol) in ethanol (20 mL) was added in one portion to an ice-cold solution of compound **6 **(3.68 g, 10 mmol) and an active methylene compound (ethyl acetoacetate, thioglycolic acid or malononitrile) (20 mmol). The mixture was stirred overnight at room temperature, the solvent evaporated *in vacuo*, and the concentrated ethanol solution then poured into cold water and the corresponding products were collected by filtration and recrystallized from ethanol. 

*Ethyl-1-[(5-{4-[ethoxycarbonyl)-5-hydroxy(1,2,3)triazol-1-yl]}-4-methyl-3-phenyl-thieno[2,3-b]thiophene-2-yl) carbonyl]-5-hydroxy(1,2,3)triazole-4-carboxylate* (**7a**). Yellow solid; yield 70%; m.p. 148 °C; IR ν_max_: 3,268 (OH), 1,713 (C=O), 1,686 (C=O) cm^-1^; ^1^H-NMR: δ 1.67 (t, 6H, 2CH_3_), 2.02 (q, 4H, 2CH_3_), 4.14 (s, 2H, CH_2_), 7.41-7.55 (m, 5H, ArH), 12.12 (s, 2H, 2OH); ^13^C-NMR: δ 10.2, 14.1, 15.5, 59.5, 61,4, 62.3, 128.2, 129.8, 135.1, 139.6, 142.2, 144.4, 147.3, 165.2, 167.7; MS *m/z *(%) 597 (M^+^+1, 88.6%), 596 (M+, 100%), 373 (99.9%), 329 (62.3%); Anal. calcd. for C_25_H_20_N_6_O_8_S_2_ (596.6): C, 50.33; H, 3.38; N, 14.09; S, 10.75. Found: C, 50.38; H, 3.40; N, 14.00; S, 10.73.

*[5-(5-Hydroxy-4-mercapto(1,2,3)triazole-1-carbonyl)-3-methyl-4-phenyl-thieno[2,3-b]thiophene-2-yl]-(5-hydroxy-4-mercapto(1,2,3)triazol-1-yl)-methanone* (**7b**). Yellow solid; yield 75%; m.p. 168 °C; IR ν_max_; 3,268 (OH), 1,682 (C=O) cm^-1^; ^1^H-NMR: δ 2.00 (s, 3H, CH_3_), 7.38–7.50 (m, 5H, ArH), 8.51(s, 2H, 2OH), 9.25(s, 2H, 2SH); ^13^C-NMR: δ 10.2, 58.5, 127.6, 129.8, 132.6, 135.1, 139.6, 142.2, 144.4, 152.3, 163.2, 167.0; MS *m/z *(%) 516 (M^+^+1, 88.6%), 515 (M+, 100%), 374 (99.9%), 227 (89.3%); Anal. calcd. for C_19_H_12_N_6_O_4_S_4_ (516.6): C, 44.17; H, 2.34; N, 16.27; S, 24.83. Found: C, 44.20; H, 2.40; N, 16.09; S, 24.77.

*[5-(5-Amino-3-cyano(1,2,3)triazole-1-carbonyl)-3-methyl-4-phenyl-thieno[2,3-b]thiophene-2-yl]-(5-amino-3-cyano(1,2,3)triazol-1-yl)-methanone* (**7c**). Yellow solid; yield 65%; m.p. 155°C; IR ν_max_: 3309 (NH_2_), 1568 (N=N), 1685 (C=O) cm^-1^; ^1^H-NMR: δ 2.04 (s, 3H, CH_3_), 4.05–427(br. s, 4H, 2NH_2_, D_2_O exchangeable), 7.34–7.45 (m, 5H, ArH); ^13^C-NMR: δ 10.2, 58.5, 127.6, 129.8, 132.6, 135.1, 139.6, 140.4, 142.2, 144.4, 152.3, 163.2, 167.0; MS *m/z *(%) 501 (M^+^+1, 88.6%), 500 (M+, 100%), 207 (22.7%), 76 (6.6%); Anal. calcd. for C_21_H_12_N_10_O_2_S_2_ (500): C, 50.39; H, 2.42; N, 27.98; S, 12.81. Found: C, 50.36; H, 2.42; N, 27.77; S, 12.67.

## 4. Conclusions

Synthesis and identification of some bis-heterocycles **3a-c, 5a-c, 6 **and **7a-c** containing thieno[2,3-b]thiophene as a base unit via the versatile, hitherto unreported 3-methyl-4-phenylthieno[2,3-b]thiophene-2,5-dicarbohydrazide (**2**) was reported.
